# *Leishmania (Sauroleishmania) tarentolae* versus pathogenic species: comparative evaluation of protease activity, glycoconjugates, resistance to complement and metabolome composition

**DOI:** 10.1590/0074-02760230243

**Published:** 2024-05-20

**Authors:** Filipe Fideles Duarte Andrade, Jéssica Gardone Vitório, Gisele André Baptista Canuto, Fernanda Freire Campos Nunes, Isabela Aurora Rodrigues, Ana Paula Martins Morais Almeida, Frederico Crepaldi Nascimento, Adriana Oliveira Costa, Tamara da Silva Vieira, Ana Carolina Carvalho Silva, Leiliane Coelho André, Célia Maria Ferreira Gontijo, Caroline Junqueira, Juliano Simões de Toledo, Ana Paula Fernandes, Rodrigo Pedro Soares

**Affiliations:** 1 Universidade Federal de Minas Gerais, Instituto de Ciências Biológicas, Departamento de Biologia Geral, Belo Horizonte, MG, Brasil; 2 Universidade Federal de Minas Gerais, Faculdade de Farmácia, Departamento de Análises Clínicas e Toxicológicas, Belo Horizonte, MG, Brasil; 3 Universidade Federal da Bahia, Instituto de Química, Departamento de Química Analítica, Salvador, BA, Brasil; 4 Fundação Oswaldo Cruz-Fiocruz, Instituto René Rachou, Grupo Biotecnologia Aplicada ao Estudo de Patógenos, Belo Horizonte, MG, Brasil

**Keywords:** Leishmania, Leishmania tarentolae, metabolomics, glycoconjugates, proteases

## Abstract

**BACKGROUND:**

*Leishmania tarentolae* is a non-pathogenic species found in lizards representing an important model for *Leishmania* biology. However, several aspects of this *Sauroleishmania* remain unknown to explain its low level of virulence.

**OBJECTIVES:**

We reported several aspects of *L. tarentolae* biology including glycoconjugates, proteolytic activities and metabolome composition in comparison to pathogenic species (*Leishmania amazonensis*, *Leishmania braziliensis*, *Leishmania infantum* and *Leishmania major*).

**METHODS:**

Parasites were cultured for extraction and purification of lipophosphoglycan (LPG), immunofluorescence probing with anti-gp63 and resistance against complement. Parasite extracts were also tested for proteases activity and metabolome composition.

**FINDINGS:**

*Leishmania tarentolae* does not express LPG on its surface. It expresses gp63 at lower levels compared to pathogenic species and, is highly sensitive to complement-mediated lysis*.* This species also lacks intracellular/extracellular activities of proteolytic enzymes. It has metabolic differences with pathogenic species, exhibiting a lower abundance of metabolites including ABC transporters, biosynthesis of unsaturated fatty acids and steroids, TCA cycle, glycine/serine/threonine metabolism, glyoxylate/dicarboxylate metabolism and pentose-phosphate pathways.

**MAIN CONCLUSIONS:**

The non-pathogenic phenotype of *L. tarentolae* is associated with alterations in several biochemical and molecular features. This reinforces the need of comparative studies between pathogenic and non-pathogenic species to elucidate the molecular mechanisms of virulence during host-parasite interactions.

*Leishmania (Sauroleishmania) tarentolae* is considered a non-pathogenic species with no medical relevance. Nevertheless, the reptile-infective *L. tarentolae* is closely related to mammalian-infective parasites, sharing a common ancestor of the subgenus *Leishmania*. Evolutionarily, *L. tarentolae* belongs to the subgenus *Sauroleishmania*, whereas the pathogenic species belong to the subgera *Leishmania*, *Viannia* and *Mundinia*. For example, *L. amazonensis*/*L. infantum*/*L. major* and *L. braziliensis* belong to the subgenus *Leishmania* and *Viannia*, respectively.^1^ ^,^ ^2^ ^,^ ^3^ ^,^ ^4^ *L. tarentolae* is transmitted by *Sergentomyia minuta* and was reported in several countries including Brazil, Portugal, Spain, France, Italy, Egypt, Tunisia, Algeria, Sudan, Togo, China, and Turkmenistan. Although its main host is the gecko *Tarentola mauritania*, its presence was also detected in humans (Brazil and Italy), dogs (Italy), snakes (China) and sandflies (Italy and Togo).^5^ ^,^ ^6^ In this context, comparative studies between the pathogenic and non-pathogenic species help to understand virulence, molecular mechanisms during host-parasite interactions, and putative *Leishmania* species interaction.^6^ ^,^ ^7^ ^,^ ^8^ A distinguished feature of *L. tarentolae* is its potential for developing recombinant proteins and vaccines against leishmaniasis.^9^ ^,^ ^10^ ^,^ ^11^ Thus, characterisation of its biological features will favour its biotechnological potential.^6^ In this context, omics approaches appear as promising tools to characterise species/strains of *Leishmania*.

Comparative genomic analysis carried out with pathogenic species and *L. tarentolae* revealed marked conservation of gene content and synteny with few specific genes.^12^ Its genome size is larger than previously expected (~35,416,496 bp) and further research is needed to properly address and compare repetitive domains.^13^ In contrast, transcriptomic, proteomic, and metabolomic approaches to characterise the molecular differences between pathogenic and lizard-infecting species are still scarce.

Among the “-omics” approaches, metabolomics provides a closer visualisation of *L. tarentolae* phenotypes.^14^ Most of the *Leishmania* metabolomic studies have been focused on the modes of action of antileishmanial drugs and resistance mechanisms.^15^ ^,^ ^16^ ^,^ ^17^ ^,^ ^18^ Besides, metabolomics was used to investigate other biological aspects of pathogenic species, including metabolic profile during stage differentiation,^19^ and to compare them.^20^

As a part of a wider study on *Leishmania* characterisation, this study aimed to access several biochemical parameters in *L. tarentolae* including glycoconjugates, proteases and resistance to complement-mediated lysis. Finally, a pilot comparative untargeted metabolomics approach explored differences among *L. tarentolae* and four pathogenic *Leishmania* species (*L. major*, *L. infantum*, *L. braziliensis,* and *L. amazonensis*). Understanding the complex mechanisms underlying the phenomenon of host-parasite interaction will help to identify targets to control or even block parasite development.

## MATERIALS AND METHODS

**Fig. 1:**
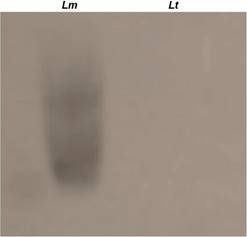
western blot of purified lipophosphoglycan (LPG). Western blot analysis of purified LPG from *Leishmania major* (*Lm*) and *Leishmania tarentolae* (*Lt*) using WIC 79.3 (1: 1,000).

**Fig. 2:**
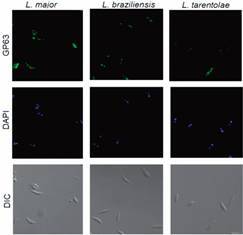
expression of glycoprotein-63 (gp63) in different *Leishmania* species. Immunofluorescence staining of *L. tarentolae*, *L. major* and *L. braziliensis* promastigotes using mAb anti-gp63 (1:500) and goat anti-mouse IgG, Alexa Fluor 488 (1:3000). DAPI (blue fluorescence) was used to stain *Leishmania* nuclei and kinetoplasts. The differential interference contrast (DIC) image of parasites can be visualised in the lower panel.

*Leishmania cultures* - A panel of five World Health Organization Reference strains of *L. tarentolae* (RTAR/DZ/1939/LV414), *L. amazonensis PH8* (IFLA/BR/67/PH8), *L. braziliensis* (MHOM/BR/75/M2904), *L. major* Friedlin clone V1 (MHOM/IL/80/Friedlin), *L. infantum* (MHOM/BR/1974/PP75) and *L. braziliensis* (MHOM/BR/94/H3227) was used. Promastigotes were grown in M199 medium at 26ºC, pH 7.4, supplemented with 10% heat-inactivated foetal calf serum (FCS), 2% male human urine, 40 mM HEPES (pH 7,5), 0.1 mM adenine, 5 mg/L hemin, 1 mg/L biotin, 100 U/mL penicillin and 100 μg/mL streptomycin. All parasite lines used in this study were genotyped by Restriction-fragment length polymorphisms analysis (RFLP-PCR) of the 70 kDa heat shock protein (HSP70) gene^21^ Supplementary data ^51^ (Figs 1-2).

*Extraction and purification of lipophosphoglycan (LPG*) - LPG extraction and purification were performed from 1x10^10^ late log-phase parasites as previously reported.^22^ Briefly, LPG was extracted after a sequential organic solvent extraction in solvent E (H_2_O/ethanol/diethyl-ether/pyridine/NH_4_OH; 15:15:5:1:0.017). The extract was dried by N_2_ evaporation and applied to a Phenyl-Sepharose column. LPG was eluted with solvent E, dried with N_2_ evaporation, and quantified by phenol-sulfuric acid method.^23^

*Indirect immunofluorescence* - Late log promastigotes (1 x 10^7^) were harvested by centrifugation (1540 ***g*** , 10 min, 4ºC), washed with phosphate-buffered saline (PBS) and fixed (2% paraformaldehyde in PBS). Then, parasites were washed three times with PBS (1540 ***g*** , 10 min, 4ºC), resuspended in 1 mL of PBS and added to poly-L-lysine coated slides. The slide was dried overnight. The wells were rehydrated twice for 15 min with PBS, followed by incubation with blocking solution (5% inactivated FCS in PBS) (1 h) at room temperature (RT). Subsequently, the primary antibody (1:500 anti-gp63) was added in blocking solution and the reaction was incubated for 2 h (RT). The wells were washed five times with PBS prior to incubation with goat anti-mouse IgG, Alexa Fluor 488 (1:3,000). The reaction was incubated (1 h, RT) and protected from light. The wells were washed five times with PBS and one drop of Vectashield solution containing DAPI was added. Images were captured by confocal microscope Nikon C2 using NIS elements software.

*Zymography assay* - Intracellular and extracellular protease activities were assessed as reported elsewhere.^24^ Briefly, 1 x 10^9^ stationary phase promastigotes were washed three times in cold PBS (1540 ***g*** , 4ºC, 10 min), resuspended in 1 mL of RPMI containing [25 mM] HEPES and incubated at 26ºC for 3 h. After incubation, the suspension was centrifuged, both pellet and supernatant were collected. The supernatant was filtered on 0.22 µM pore membrane filters. The pellet was washed three times in cold PBS and resuspended in lysis buffer (1% of Triton X-100, [10 mM] Tris-HCl, pH 6.8). Both fractions were incubated in the presence/absence of 5 mM of ethylene diamine tetra acetic acid (EDTA), a metalloprotease inhibitor and phenylmethanesulfonylfluoride (PMSF), a serine protease inhibitor. Samples (10 μg) were resolved by 10% sodium dodecyl sulphate-polyacrylamide gel electrophoresis (SDS-PAGE) copolymerised with 0.1% gelatin. Gels were washed twice for 30 min in renaturation solution (2% Triton-X 100) and were incubated for 48 h at 37ºC in reaction buffer ([50 mM] Tris-HCl, [10 mM] CaCl_2_, pH 5.5). Gels were stained with 0.05% Coomassie Blue G-250, 10% acetic acid and 30% methanol.

*Complement-mediated lysis* - These experiments were performed according to adaptations on the protocol described by Späth et al.^25^ The pool of human serum was obtained from our cryobank. Prior to the experiment, 1 x 10^7^ logarithmic or stationary phase promastigotes were centrifuged for 5 min, 50 ***g*** , 4ºC, to minimise the proportion of rosettes and non-viable cells. After, parasites were collected from the supernatant by centrifugation at 1540 ***g*** at 4ºC for 10 min and were washed three times with cold PBS. They were resuspended in 500 µL of Dulbecco’s Modified Eagle Medium (DMEM) without FCS, containing 40 μg of propidium iodide (PI) and 4% human serum (30 min, 26ºC). Then, parasites were washed in cold PBS, resuspended in 300 µL of PBS containing 1% formaldehyde, and the fluorescence was assessed by flow cytometer BD LSR Fortessa™. The reactions were performed in the presence of non-stained controls (without PI), negative controls (without human serum), and positive control (parasites heated at 100ºC for 30 min in the presence of PI) to assist gate construction. The results were analysed using FlowJo^®^ v10.

*Comparative metabolomics by Gas chromatography-mass spectrometry (GC-MS), metabolite extraction and sample treatment* - Parasites (4x10^7^) were collected by centrifugation (1540 ***g*** , 10 min, 4ºC) and washed three times in 1 mL of cold PBS. Cultures were quenched by rapid decrease of temperature in dry ice/ethanol bath. Cell pellets were resuspended in 300 µL of extraction mixture (methanol, water, and chloroform, 3:1:1 v/v/v), followed by four cycles of freeze/thaw. After centrifugation (16,000 ***g*,** 10 min, 4ºC), 200 µL of the supernatant were collected and transferred to a GC vial with insert and evaporated in Speedvac. For methoximation, 10 µL of O-methoxyamine hydrochloride in pyridine (15 mg/mL) were added to each vial and vortexed vigorously for 5 min (16 h, RT). For silylation, 10 µL of BSTFA with 1% TMCS were added to the samples and vortexed for 5 min and incubated at 70ºC for 1 h. Finally, 100 µL of heptane containing C18:0 methyl ester (10 mg/L) was added as internal standard and vortexed for 2 min prior to GC analysis. Two blank samples were prepared using same analytical procedures. Quality control (QCs) samples were prepared by pooling equal volumes of each sample and were analysed throughout the run, after each five samples.

*GC-MS instrumentation* - Two µL of derivatised samples were injected in GC instrument (Agilent 7890A) coupled to mass spectrometer with triple-Axis detector (Agilent 5975C) in split mode using Agilent GC autosampler 80 for injection. Separation was achieved using an DB5-MS column (30 m length, 0.25 mm internal diameter, with a 0.25 µm thickness film consisted of 95% dimethylpolysiloxane/5% diphenylpolysiloxane) (Agilent Technologies^®^). Helium was used as the carrier gas with a flow rate set at 1 mL/min. The injector temperature was held at 250ºC and the split ratio was 1:10. The column was initially maintained at 60ºC for 1 min after injection, then temperature was gradually increased at the rate of 10ºC/min to a final temperature of 325ºC. Finally, it was cooled down after analysis for 10 min. The run time was 37.5 min. The temperatures of the injector, transfer line, filament source and quadruple were held at 250ºC, 280ºC, 230ºC and 150ºC, respectively. The quadrupole detector (5975 inert MSD, Agilent) was controlled by MSD ChemStation E.02.02.1431. The electron ionisation source was operated at 70 eV. The mass spectrometer operated in scan mode over a mass range m/z 50-600 at a rate of 2.7 scan/s. The samples were analysed in one randomised run to reduce the effects of variations in equipment performance.^26^

*Data treatment* - Data processing was performed in XCMS (version 1.24.1)^27^ executed in R platform (version 3.2.2). Data extraction was done by the matched filter method. The following parameters were used to detect the peaks: peak width (fwhm) = 4, signal-to-noise ratio (snthresh) = 1.5, maximum number of peaks per extracted ion chromatogram (max) = 30. Peaks were grouped based on bandwidth correction (bw) = 2 and the width of the overlapped bands of m/z (mzwid) = 0.25. The default “retcor” method was applied with nonlinear alignment and smoothing degree of polynomial regression adjustment (span) = 0.5 to correct the retention time. The “fillPeaks” tool was used to remove missing values. The data matrix was normalised by the median of intensities, followed by internal standard normalisation (C18:0 methyl stearate) before statistical analyses. AMDIS (version 2.71) was used for identification of co-eluted compounds based on the retention index and retention time analysis using Fiehn RLT Library (FiehnLib).^28^

*Statistical analysis* - Multivariate statistical analyses were performed on Pareto scaled data. Principal components analysis (PCA) and partial least square discriminant analysis (PLS-DA) were carried out using SIMCA P+ 14.1. Univariate pairwise comparisons were performed using the Statistica version 10, using either Student’s t test or Mann Whitney U test for independent data, according to normality tests (Kolmogorov-Smirnov, Lilefors and Shapiro-Wilk). A p-value smaller than 0.05 was considered significant. The heatmap was built in MetaboAnalyst version 4.0. Pathway enrichment analysis was performed on MBROLE 2.0 (http:// csbg.cnb.csic.es/mbrole2/).

## RESULTS

*LPG is absent in L. tarentolae promastigotes* - To investigate whether differences in LPG expression could be associated to the avirulent phenotype of *L. tarentolae,* we assessed this glycoconjugate using the mAb WIC 79.3, specific for terminal Gal(β1,3) sidechains. In contrast to *L. major*, no reactivity of *L. tarentolae* was detected (Fig. 1).

**Fig. 3:**
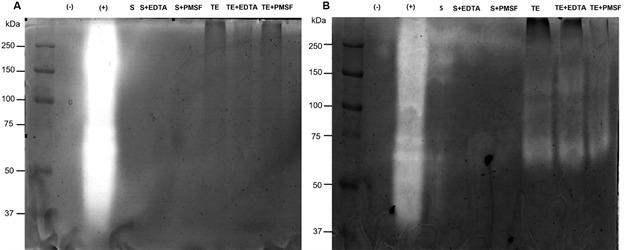
evaluation of intracellular and secreted proteases activity of *Leishmania tarentolae* and *L. amazonensis*. (A) The activity of secreted and intracellular proteases of *L. tarentolae* LV-414 and (B) *L. amazonensis* PH8 (10 μg/lane) were assessed after incubation of gels in reaction buffer in the presence/absence of 5 mM EDTA (metallo-protease inhibitor) or PMSF (serine-protease inhibitor). *Acanthamoeba castellanni* genotype T4 extract (10 μg/lane) was used as a positive control and deionised water was used as a negative control. (-): negative control; (+): positive control; S: supernatant; TE: total extract; EDTA: ethylenediamine tetra acetic acid; PMSF: phenylmethylsulfonylfluoride.

*Proteases profile in L. tarentolae* - Since *L. tarentolae* did not express LPG, our next step was to search for another major glycoconjugate (gp63) by immunofluorescence. Promastigotes of *L. tarentolae*, *L. major*, and *L. braziliensis* were recognised by mAb anti-gp63 (Fig. 2). Nevertheless, gp63 expression seemed to be lower in *L. tarentolae* compared to pathogenic species (Fig. 2). Next, to evaluate if *L. tarentolae* had other active proteases to compensate the scarceness of gp63, intracellular and secreted enzymes were subjected to zymography. As expected, the positive control of *A. castellani* had strong proteolytic activity (Fig. 3A-B). No proteolytic activity was detected in *L. tarentolae* (culture supernatant and cellular extracts) (Fig. 3A). In contrast, proteolytic activities between 100-250 kDa and 50-150 kDa, were respectively observed in the culture supernatant and cellular extracts of *L. amazonensis* promastigotes (Fig. 3B). The proteolytic profile exhibited by *L. amazonensis* suggested the presence of at least one intracellular and one secreted serine protease, and two other proteases that could not be identified using protease inhibitors (Fig. 3B).

**Fig. 4:**
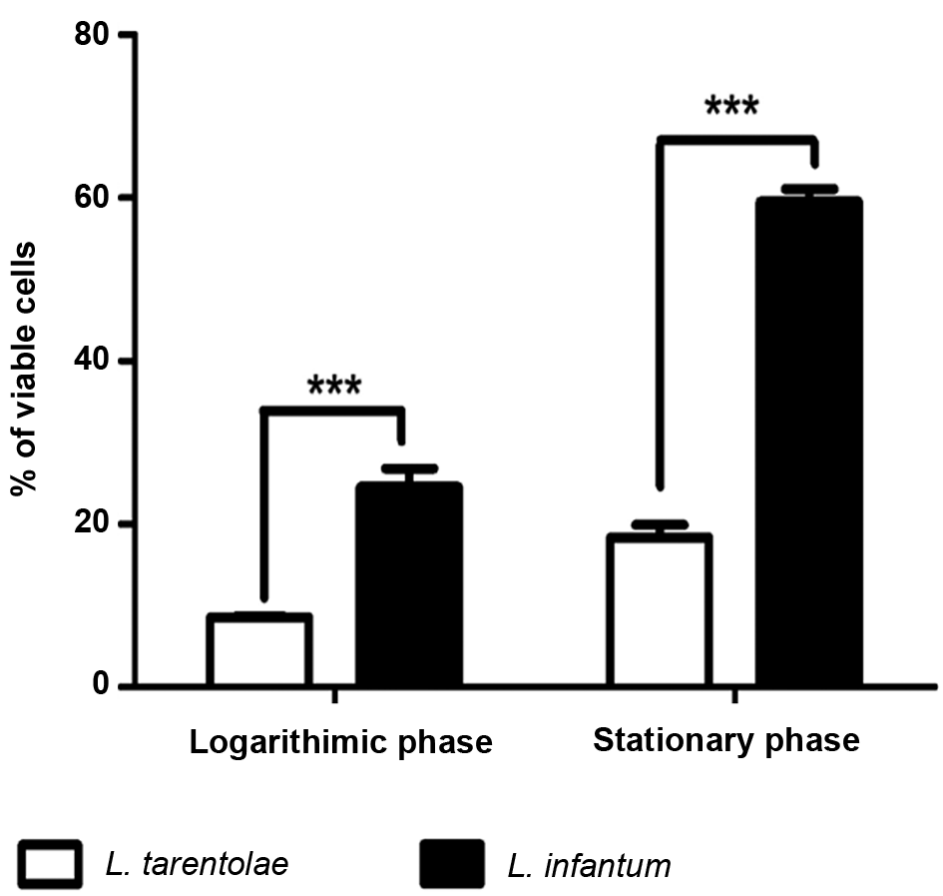
*Leishmania tarentolae* susceptibility to the complement system. The graph was constructed using the percentage of viable cells, comparing the susceptibility of *L. tarentolae* LV-414 and *L. infantum* PP75 to complement-mediated lysis during logarithmic and stationary phase. The data were analysed by Two-way analysis of variance (ANOVA) with Bonferroni post-test. ****: p-value < 0.01.

*Leishmania tarentolae is highly sensitive to human serum* - Since the combined deficiency of LPG and proteolytic activity could render *L. tarentolae* more susceptible to innate immune responses, we evaluated the susceptibility of these parasites to the human complement system. Promastigotes (logarithmic and stationary) of *L. tarentolae* are significantly more susceptible to complement-mediated lysis than *L. infantum* ones (Fig. 4). As expected, the resistance of *L. infantum* promastigotes to complement-mediated lysis increased significantly during stationary phase.

**Fig. 5:**
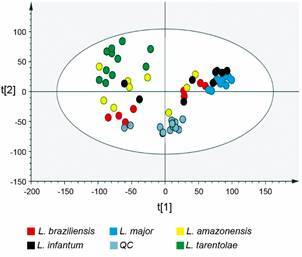
principal component analysis (PCA) for five species of *Leishmania* (*L. amazonensis*, *L. braziliensis*, *L. infantum*, *L. major*, and *L. tarentolae*) and quality controls. Quality parameters: explained variance R^2^ = 0.738 and predicted variance Q^2^ = 0.630.

*Metabolic profile between pathogenic and non-pathogenic species* - Using GC-MS, metabolic differences among *L. tarentolae* and four pathogenic species were assessed. Forty-seven metabolites were identified and their respective physicochemical properties [Supplementary data (Table I)]. Out of the 47 metabolites, 10 were not detected in *L. tarentolae*, including uridine-5-monophosphate, ribose-5-phosphate, ornithine, lysine, hypoxanthine, glucose-6-phosphate, citric acid, adenosine-5-monophosphate, 6-phosphogluconic acid, and phosphoenolpyruvic acid [Supplementary data (Table I)]. The appropriate quality of the analytical procedures was demonstrated by clustering QCs in a well-defined area of a PCA plot (Fig. 5).

**Fig. 6:**
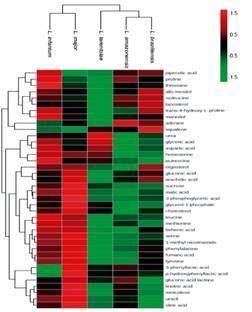
hierarchical clustering analysis (HCA) and heatmap showing the metabolic profile of five *Leishmania* species. Following a log-transformation (base 2) of the mean ionic abundance, the relative level of each compound across the species is represented in the lines. The intensity of each metabolite is indicated by the colour code, ranging from -1.5 (green) to 1.5 (red). The dendrogram displayed on the top of the x-axis indicates clear differences in the metabolic profile of the pathogenic species and *L. tarentolae*. The dendrogram on the left shows the clustering of metabolites based on similarity in intensity profile. The following parameters were used in the construction of dendrograms: Euclidean distance; complete-linkage clustering method. MetaboAnalyst 4.0 was used to generate the Figure.

A heatmap was built to allow an overview of the differences in the metabolic profile of the species of *Leishmania* (Fig. 6). Only metabolites that were found in all species, but with different abundances were included in the construction of this graph. Hierarchical clustering analysis (HCA) revealed marked differences, clearly distinguishing each *Leishmania* species. The metabolic profile of *L. tarentolae* clearly differs from the profile of the pathogenic species.

To identify a metabolic signature in each species, a PLS-DA was generated. Again, *L. tarentolae* samples differed from those of pathogenic species [Supplementary data (Fig. 3A)]. R2X, R2Y, and Q2X quality parameters showed scores above 0.8 that were validated by a permutation test with 100 iterations [Supplementary data (Fig. 3B)]. PLS-DA [Supplementary data (Fig. 4)] also compared species in pairs and used the variable importance in projection (VIP). This enables us to identify variables that most significantly contributed to group discrimination in these models [Supplementary data (Table II)].

To identify pathways that diverge among *L. tarentolae* and the other pathogenic species, the significantly altered metabolites between each species pair were used for pathway enrichment analysis (Table I). The metabolites that enriched each pathway are listed [Supplementary data (Table III)]. Clear differences among the stationary-phase promastigotes of *L. tarentolae* and the pathogenic species were detected. Steroid biosynthesis and pentose-phosphate pathways (PPP) differed among pathogenic and non-pathogenic species. Depending on *Leishmania* species, *L. tarentolae* differed in several metabolic pathways including biosynthesis of unsaturated fatty acids and TCA cycle (*L. major*/*L. infantum*); glyoxylate and dicarboxylate metabolism (*L. amazonensis*/*L. major*) and ABC transporters and glycine, serine, and threonine metabolism (*L. amazonensis*). Using VIP values higher than 1.0 and p-value lower than 0.05, we identified metabolic signatures able to differentiate *L. tarentolae* from pathogenic species (Tables II-III). In Table II, a selection of 10 metabolites not found in *L. tarentolae* is provided. In Table III, the metabolites that most significantly contributed to differences between *L. tarentolae* and pathogenic species are identified. Finally, data on Supplementary data (Table III) summarises the relevant metabolic pathways enriched in *L. tarentolae* versus pathogenic species.

**TABLE I:** KEGG enrichment analysis for the statistically significant metabolites between *Leishmania tarentolae* and pathogenic species

Enriched metabolic pathways	*Lt*/ *Li*		*Lt*/ *Lm*		*Lt/ La*		*Lt/ Lb*	
	p-value	FDR	p-value	FDR	p-value	FDR	p-value	FDR
ABC transportes	-	-	-	-	*	*	-	-
Biosynthesis of unsaturated fatty acids	*	*	*	*	-	-	-	-
Citrate cycle (TCA cycle)	*	*	*	*	-	-	-	-
Glycine, serine, and threonine metabolism	-	-	-	-	*	*	-	-
Glyoxylate and dicarboxylate metabolism	-	-	*	*	*	*	-	-
Pentose-phosphate pathway	*	*	*	*	*	*	*	*
Steroid biosynthesis	*	*	*	*	*	*	*	*

*: p-value and FDR-p < 0.05; -: not significantly enriched in each pair. FDR: false discovery rate corrected p-value; *Lm*: *L. major Friedlin*; *Lt*: *L. tarentolae* LV-414; *Lb*: *L. braziliensis* M2904; *Li*: *L. infantum* PP75; *La*: *L. amazonensis* PH8; KEGG: Kyoto Encyclopedia of Genes and Genomes*.*

**TABLE II:** Metabolites identified by gas chromatography-mass spectrometry (GC-MS) that were not detected in *Leishmania tarentolae*

Metabolites	Molecular formula	Monoisotopic mass	Biochemical categories
Monosaccharide phosphate (6-phosphogluconic acid)	C_6_H_13_O_10_P	276.025	Carbohydrates
Adenosine-5-Monosphosphate	C_10_H_14_N_5_O_7_P	347.063	Purines, pyrimidines, and conjugates
Citric acid	C_6_H_8_O_7_	192.027	Organic acids and derivatives
Hexose phosphate (Glucose-6-phosphate)	C_6_H_13_O_9_P	260.030	Carbohydrates
Hypoxanthine	C_5_H_4_N_4_O	136.039	Purines, pyrimidines, and conjugates
Lysine	C_6_H_14_N_2_O_2_	146.106	Amino acids, peptides, and conjugates
Phosphoenolpyruvic acid	C_3_H_5_O_6_P	167.982	Organophosphate
Pentose-phosphate (Ribose-5-phosphate)	C_5_H_11_O_8_P	230.019	Carbohydrates
Ornithine	C_5_H_12_N_2_O_2_	132.090	Amino acids, peptides, and conjugates
Uridine-5-monophosphate	C_9_H_13_N_2_O_9_P	324.036	Purines, pyrimidines, and conjugates

**TABLE III:** Metabolites with statistical significance among *Leishmania tarentolae* and pathogenic species

Metabolite	*Lt x La*		*Lt x Lb*		*Lt x Li*		*Lt x Lm*	
	p-value	VIP	p-value	VIP	p-value	VIP	p-value	VIP
6-phosphogluconic acid	3.89E-03	2.23	9.50E-04	2.35	1.97E-04	2.09	1.97E-04	2.04
Adenosine-5-monophosphate			4.02E-02	1.61	1.35E-02	1.41	1.97E-04	1.81
Allo-inositol	1.97E-04	1.31	1.97E-04	1.64	1.97E-04	1.14	1.97E-04	
Citric acid	4.02E-02	1.51	4.02E-02	1.49	1.35E-02	1.49	1.97E-04	1.73
Ergosterol	1.97E-04	1.80	1.97E-04	1.84	1.97E-04	1.34	1.97E-04	1.21
Glucose-6-phosphate	1.35E-02	1.92		1.15	3.89E-03	1.36	1.97E-04	1.75
Hypoxanthine					4.02E-02	1.34	3.89E-03	1.39
Lanosterol	2.09E-03	1.19	1.97E-04	1.47	1.09E-03		8.29E-04	
Melezitose	1.97E-04	1.31			4.74E-04		1.97E-04	1.31
Ornithine			4.02E-02	1.48	1.35E-02	1.49	1.97E-04	1.59
Phenylalanine	4.94E-03		2.37E-03	1.17	1.97E-04	1.04	1.97E-04	1.11
Proline	1.97E-04	1.74	1.97E-04	1.19	1.97E-04	1.49	1.97E-04	
Ribose-5-phosphate	1.35E-02	1.81	4.02E-02	1.47	3.89E-03	1.46	1.97E-04	1.72
Threonine	1.32E-07	1.05	1.96E-05		1.97E-04	1.17	4.07E-06	
Uridine-5-monophosphate	1.35E-02	1.69	9.50E-04	1.97	3.89E-03	1.31	1.97E-04	1.72

*Lm*: *L. major* Friedlin; *Lt*: *L. tarentolae* LV-414; *Lb*: *L. braziliensis* M2904; *Li*: *L. infantum* PP75; *La*: *L. amazonensis* PH8; VIP: variable importance in projection.

## DISCUSSION

Nowadays, the search for molecular and biochemical parameters that could distinguish pathogenic from non-pathogenic species emerges as an alternative for understanding the complexity during host-parasite interaction. *L. tarentolae* is a non-pathogenic species and despite the recent biotechnological advances, many aspects of its biology remain unknown compared to the pathogenic species. Those included existence of metacyclic forms, LPG presence and the composition and variability of its proteome and metabolome.

GP63 and LPG are the major *Leishmania* glycoconjugates playing numerous roles during host-parasite interactions.^29^ ^,^ ^30^ ^,^ ^31^ Importantly, these molecules contribute for protection against complement-mediated lysis, a pivotal mechanism to promote infection establishment.^29^ ^,^ ^31^ Preliminary reports based on an immunofluorescence approach using CA7AE, suggested that LPG is absent in this lizard-infecting species.^32^ Here, we further demonstrated that LPG is absent in *L. tarentolae*, using mAb WIC79.3. Genome sequencing did not identify differences in *L. tarentolae* sequences that could support LPG absence in this species. Only a reduced number of copies or absence of genes involved in modification and addition of the glycoconjugate sidechains was found.^12^ This feature was also reported for monoxenic trypanosomatids, e.g *Leptomonas pyrrhocoris* that do not express LPG, despite the fact that they have genomic sequences coding for side-chains galactosyl transferases (*SCGs*).^33^ Not all SCGs are functional even pathogenic species as *L. major* ^34^ ^,^ ^35^ ^,^ ^36^ and this may be the case of *L. tarentolae*.

Different from LPG, a non-proteolytic gp63 is present in *L. tarentolae* surface and anchored to its extracellular vesicles.^37^ ^,^ ^38^ Corroborating the other studies, we observed the presence of gp63 in *L. tarentolae*,^12^ ^,^ ^37^ ^,^ ^38^ but at reduced levels in comparison to pathogenic species. The high variability in gp63 sequence in *L. tarentolae* demonstrated by genomic studies^12^ may hinder its reactivity against mAb anti-gp63 used here. Consistent with these observations, strains of *L. braziliensis* display variable reactivity against mAb anti-gp63, showing different isoforms and levels of expression.^39^

Our analysis of *L. tarentolae* identified the absence of proteolytic activity. This may be a result of the low concentration and/or with reduced activity of proteases. In this context, experiments employing azocasein as substrate demonstrated very low proteolytic activity of *L. tarentolae* compared to those of *Trypanosoma* spp.^40^ Due to the combined absence of LPG and the putative reduced proteolytic activity of gp63 and other proteases, we demonstrated that *L. tarentolae* is significantly more sensitive to complement-mediated lysis than *L. infantum*. Considering that the complement system is one of the first-line host’s defences, this lizard-infecting species is less capable to survive in mammalian hosts.^41^ ^,^ ^42^

Previous studies evidenced increased glucose consumption during the stationary phase of pathogenic species, identifying higher expression of enzymes implicated in glycolysis and TCA cycle. Alterations in glucose metabolism might arise from the energetic demand during the differentiation from procyclic to metacyclic promastigotes.^43^ Interestingly, the levels of several glycolysis and TCA cycle intermediates either were reduced or not detected in *L. tarentolae* in our metabolomics approaches. Notably, no abundance of glucose-6-phosphate and citric acid were noticed in the non-pathogenic species. These findings could be associated with the higher copy numbers of genes coding for glucokinase-1 and citrate synthase in *L. tarentolae*.^12^ These could contribute to increased production of those enzymes, leading to a marked consumption of carbohydrates *via* glycolysis and TCA cycle in these parasites. As a result, high promastigote growing leads to diminished availability of these substrates in the stationary phase.

*Leishmania* is not capable of synthesising cholesterol and should acquire this compound from the culture medium or their hosts.^44^ Thus, the differences detected in cholesterol concentrations among the five studied species may reflect variations on the expression of proteins involved in its absorption from the environment. In contrast to cholesterol, ergosterol, lanosterol, and squalene are *de novo* synthesised by parasites through the mevalonate acid pathway.^43^ ^,^ ^45^ Interestingly, the five species exhibited contrasting levels of these sterols. For instance, *L. tarentolae* and *L. amazonensis* displayed a low abundance of ergosterol, while *L. braziliensis*, *L. infantum*, and *L. major* did not. The intracellular levels of sterols may reflect not only different enzymatic activity of the mevalonate pathway, but also a membrane remodelling process during differentiation into stationary-phase promastigotes. Indeed, during *in vitro* cultivation of *Leishmania* the levels of sterols undergo drastic changes in concentration.^44^ The levels of ergosterol isomers decrease drastically in stationary-phase promastigotes compared to logarithmic-phase ones.^44^ Moreover, the level of these compounds is approximately twice as low in purified metacyclic promastigotes than in stationary-phase forms.^44^ These dynamic changes in sterol levels during parasite development suggest a possible role of these compounds in the virulence of *Leishmania*.^44^ Therefore, our results might be related to differences in metacyclogenesis rates and requirements for membrane remodelling among the studied species.

In *Leishmania*, the pyrimidine pool is synthesised *de novo* by parasites and obtained from their hosts.^46^ Uracil phosphoribosyltransferase (UPRT) is the enzyme responsible for the incorporation of preformed exogenous pyrimidines into the parasite nucleotide pool. The precursors are converted to uracil and then phosphoribosylated to uridine monophosphate by this enzyme.^47^ The lower levels of uracil, in comparison to *L. major* and *L. infantum*, and to a lesser extent to *L. amazonensis*, and the absence of uridine 5-monophosphate found in *L. tarentolae* may correlate with the lower copy numbers of UPRT coding sequence in the genome of this species.^12^ In the literature, uracil and uridine have several functions related to pyrimidines pathways. Their blockage and/or impairment may affect RNA and DNA replication and hinder *L. donovani* growth and survivorship in the host.^39^ In the phagolysosomes of macrophages, *Leishmania* parasites confront scarcity of several nutrients and the presence of significant amounts of compounds. Those include purine precursors and pyrimidines that may facilitate the differentiation into amastigotes.^19^ One could hypothesise that the lower abundance of these compounds in *L. tarentolae* leads to its limited ability to obtain these nutrients, and to differentiate them into amastigotes. This will make them more susceptible to the harsh macrophage intracellular environment.

The use of chemically defined mediums for parasite cultivation revealed the nutritional aspects of *L. tarentolae*.^48^ In this context, L-proline constitutes a major energetic source for this lizard-infecting species.^48^ Curiously, our metabolomic analysis demonstrated that proline is reduced in *L. tarentolae* during stationary phase. This finding suggests an extensive usage of this compound during growth. Moreover, *L. tarentolae* exhibited the lowest levels of serine among the studied species. Notably, this amino acid is implicated in the biosynthesis of phospho- and sphingolipids,^49^ which are both critical for *Leishmania* virulence.^50^ Therefore, variations in amino acid concentrations may be associated to the pathogenicity of *Leishmania* and further studies are needed to clarify their molecular roles during infection.

In conclusion, we were able to describe the biology, several metabolites and metabolic pathways potentially involved in the phenotypic and molecular diversity of *Leishmania*. The combined deficiency of LPG, activity of gp63 and proteases in *L. tarentolae*, make this species more susceptible to complement-mediated lysis*.* Furthermore, metabolomic analysis revealed significant differences in the absence/abundance of certain metabolites among *L. tarentolae* and pathogenic species. Many of them are implicated in metacyclogenesis, infectivity, and ability to proliferate within the host cell. The present study corroborates the importance of comparative studies among pathogenic and non-pathogenic species to uncover novel aspects related to *Leishmania* virulence.
