# Supplementary material for: Leishmania (Sauroleishmania) tarentolae versus pathogenic species: comparative evaluation of protease activity, glycoconjugates, resistance to complement and metabolome composition
Source: Mem Inst Oswaldo Cruz. 2024 May 20;119:e230243. doi: 10.1590/0074-02760230243 (PMC11111114; doi:10.1590/0074-02760230243)
Supplement: Supplementary file 1 [file 1678-8060-mioc-119-e230243-s.pdf]

TABLE I

Metabolites identified by gas chromatography-mass spectrometry (GC-MS)

| Metabolites | Molecular formula | Monoisotopic mass | Biochemical categories | Fold changes | | | |
|---|---|---|---|---|---|---|---|
| | | | | *Lt/ Lb* | *Lt/ Lm* | *Lt/ La* | *Lt/ Li* |
| 1-methylnicotinamide | $C_7H_9N_2O$ | 137.071 | Heterocyclic aromatic compounds | 0.837 | 0.685 | 0.914 | 0.733 |
| 3-phenyllactic acid | $C_9H_{10}O_3$ | 166.063 | Organic acids and derivatives | 0.928 | 0.747 | 0.866 | 1.547 |
| Sugar acid (3-phosphoglyceric acid) | $C_3H_7O_7P$ | 185.993 | Carbohydrates | 0.968 | 0.780 | 1.014 | 0.888 |
| Monosaccharide phosphate (6-phosphogluconic acid) | $C_6H_{13}O_{10}P$ | 276.025 | Carbohydrates | * | * | * | * |
| Adenine | $C_5H_5N_5$ | 135.054 | Purines, pyrimidines and conjugates | 0.968 | 0.979 | 0.954 | 0.987 |
| Adenosine-5-Monosphosphate | $C_{10}H_{14}N_5O_7P$ | 347.063 | Purines, pyrimidines and conjugates | * | * | 0.000 | * |
| Cyclohexanol (Allo-inositol) | $C_6H_{12}O_6$ | 180.063 | Carbohydrates | 0.747 | 0.820 | 0.838 | 0.786 |
| Arachidic acid | $C_{20}H_{40}O_2$ | 312.303 | Fatty acids and conjugates | 0.887 | 0.861 | 0.929 | 0.926 |
| Aspartic Acid | $C_4H_7NO_4$ | 133.038 | Amino acids, peptides and conjugates | 1.116 | 1.065 | 1.223 | 0.967 |
| Behenic acid | $C_{22}H_{44}O_2$ | 340.334 | Fatty acids and conjugates | 0.954 | 0.895 | 1.004 | 0.903 |
| Cholesterol | $C_{27}H_{46}O$ | 386.355 | Lipids and sterols | 1.015 | 0.932 | 1.089 | 0.978 |
| Citric acid | $C_6H_8O_7$ | 192.027 | Organic acids and derivatives | * | * | * | * |
| Ergosterol | $C_{28}H_{44}O$ | 396.339 | Lipids and sterols | 0.610 | 0.619 | 2.685 | 0.655 |
| Fumaric acid | $C_4H_4O_4$ | 116.011 | Organic acids and derivatives | 0.851 | 0.743 | 0.964 | 0.805 |
| Sugar acid (Gluconic acid) | $C_6H_{12}O_7$ | 196.058 | Carbohydrates | 0.857 | 0.808 | 0.899 | 0.865 |
| Gluconolactone (Gluconic acid lactone) | $C_6H_{10}O_6$ | 178.048 | Carbohydrates | 1.380 | 0.840 | 0.903 | 0.944 |
| Hexose phosphate (Glucose-6-phosphate) | $C_6H_{13}O_9P$ | 260.030 | Carbohydrates | * | * | * | * |
| Sugar acid (Glyceric acid) | $C_3H_6O_4$ | 106.027 | Carbohydrates | 1.138 | 1.107 | 1.201 | 1.021 |
| Glycerol-1-phosphate | $C_3H_9O_6P$ | 172.014 | Glycerophosphates | 1.136 | 0.819 | 1.189 | 0.976 |
| Hypoxanthine | $C_5H_4N_4O$ | 136.039 | Purines, pyrimidines and conjugates | 0.000 | * | 0.000 | * |
| Homoserine | $C_4H_9NO_3$ | 119.058 | Amino acids, peptides and conjugates | 1.192 | 0.988 | 1.228 | 0.950 |
| Lanosterol | $C_{30}H_{50}O$ | 426.386 | Lipids and sterols | 0.435 | 0.511 | 0.506 | 0.509 |
| Leucine | $C_6H_{13}NO_2$ | 131.095 | Amino acids, peptides and conjugates | 0.916 | 0.792 | 0.920 | 0.753 |
| Linoleic acid | $C_{18}H_{32}O_2$ | 280.240 | Fatty acids and conjugates | 1.043 | 0.637 | 0.798 | 0.811 |
| Lysine | $C_6H_{14}N_2O_2$ | 146.106 | Amino acids, peptides and conjugates | 0.000 | * | 0.000 | * |
| Malic acid | $C_4H_6O_5$ | 134.022 | Organic acids and derivatives | 0.989 | 0.905 | 1.017 | 0.936 |
| Sugar alcohol (Mannitol / xylitol[1]) | $C_6H_{14}O_6$ | 182.079 | Carbohydrates | 0.913 | 0.914 | 0.936 | 0.866 |
| Oligosaccharide (Melezitose) | $C_{18}H_{32}O_{16}$ | 504.169 | Carbohydrates | 0.938 | 0.454 | 0.621 | 0.589 |
| Methionine | $C_5H_{11}NO_2S$ | 149.051 | Amino acids, peptides and conjugates | 0.982 | 0.832 | 0.933 | 0.822 |
| Oleic acid | $C_{18}H_{34}O_2$ | 282.256 | Fatty acids and conjugates | 1.044 | 0.778 | 0.922 | 0.877 |
| Phosphoenolpyruvic acid | $C_3H_5O_6P$ | 167.982 | Organophosphate | 0.000 | * | 0.000 | * |
| Phenylalanine | $C_9H_{11}NO_2$ | 165.079 | Amino acids, peptides and conjugates | 0.775 | 0.673 | 0.882 | 0.746 |
| Ornithine | $C_5H_{12}N_2O_2$ | 132.090 | Amino acids, peptides and conjugates | * | * | 0.000 | * |
| p-hydroxyphenyllactic acid | $C_9H_{10}O_4$ | 182.173 | Organic acids and derivatives | 1.255 | 0.746 | 1.041 | 1.675 |
| Pipecolic acid | $C_6H_{11}NO_2$ | 129.079 | Amino acids, peptides and conjugates | 0.756 | 1.142 | 0.743 | 0.628 |
| Proline | $C_5H_9NO_2$ | 115.063 | Amino acids, peptides and conjugates | 0.802 | 0.857 | 0.710 | 0.645 |
| Putrescine | $C_4H_{12}N_2$ | 88.100 | Nitrogenous compounds and polyamines | 1.176 | 1.015 | 1.079 | 0.919 |
| Pentose-phosphate (Ribose-5-phosphate) | $C_5H_{11}O_8P$ | 230.019 | Carbohydrates | * | * | * | * |
| Serine | $C_3H_7NO_3$ | 105.043 | Amino acids, peptides and conjugates | 0.936 | 0.786 | 0.976 | 0.835 |
| Squalene | $C_{30}H_{50}$ | 410.391 | Lipids and sterols | 0.924 | 1.114 | 1.027 | 1.074 |
| o-glycosyl compound (Sucrose) | $C_{12}H_{22}O_{11}$ | 342.116 | Carbohydrates | 1.019 | 0.914 | 1.027 | 0.963 |
| Threonine | $C_4H_9NO_3$ | 119.058 | Amino acids, peptides and conjugates | 0.889 | 0.903 | 0.861 | 0.739 |
| Trans-4-hydroxy-L-proline | $C_5H_9NO_3$ | 131.058 | Amino acids, peptides and conjugates | 0.774 | 0.860 | 0.904 | 0.750 |
| Tyrosine | $C_9H_{11}NO_3$ | 181.074 | Amino acids, peptides and conjugates | 0.789 | 0.639 | 1.015 | 0.699 |
| Uracil | $C_4H_4N_2O_2$ | 112.027 | Purines, pyrimidines and conjugates | 1.245 | 0.704 | 0.908 | 0.786 |
| Urea | $CH_4N_2O$ | 60.032 | Nitrogenous compounds and polyamines | 1.014 | 1.010 | 1.023 | 1.008 |
| Uridine-5-monophosphate | $C_9H_{13}N_2O_9P$ | 324.036 | Purines, pyrimidines and conjugates | * | * | * | * |

Fold changes were calculated using the mean ionic abundance. '0' means that the metabolite is absent in both species of the given comparison; '*' indicates that the metabolite is absent only in *Leishmania tarentolae*. [1]It is not possible to differentiate isomers with close retention time using only GC-MS. *Lm*: *L. major Friedlin*; *Lt*: *L. tarentolae* LV-414; *Lb*: *L. braziliensis* M2904; *Li*: *L. infantum* PP75; *La*: *L. amazonensis* PH8.

CC BY

TABLE II
Metabolites with statistical significance among *Leishmania tarentolae* and pathogenic species

| | *Lt x La* | | *Lt x Lb* | | *Lt x Li* | | *Lt x Lm* | |
|---|---|---|---|---|---|---|---|---|
| Metabolite | p-value | VIP | p-value | VIP | p-value | VIP | p-value | VIP |
| 1-methyl nicotinamide | | | 4.82E-02 | | 4.74E-04 | | 1.97E-04 | |
| 3-phenyllactic acid | | | | | 2.69E-03 | | 1.97E-04 | |
| 3-phosphoglyceric acid | | | | | | | 2.66E-04 | |
| 6-phosphogluconic acid | 3.89E-03 | 2.23 | 9.50E-04 | 2.35 | 1.97E-04 | 2.09 | 1.97E-04 | 2.04 |
| Adenine | 1.28E-02 | | 1.85E-02 | | | | | |
| Adenosine-5-monophosphate | | | 4.02E-02 | 1.61 | 1.35E-02 | 1.41 | 1.97E-04 | 1.81 |
| Allo-inositol | 1.97E-04 | 1.31 | 1.97E-04 | 1.64 | 1.97E-04 | 1.14 | 1.97E-04 | |
| Arachidic acid | | | 3.34E-02 | | 5.53E-03 | | 1.97E-04 | |
| Aspartic acid | 5.72E-09 | 1.29 | 6.89E-04 | | | | 2.01E-03 | |
| Behenic acid | | | | | 2.43E-04 | | 1.88E-05 | |
| Cholesterol | 3.89E-03 | | | | 2.27E-02 | | 3.22E-06 | |
| Citric acid | 4.02E-02 | 1.51 | 4.02E-02 | 1.49 | 1.35E-02 | 1.49 | 1.97E-04 | 1.73 |
| Ergosterol | 1.97E-04 | 1.80 | 1.97E-04 | 1.84 | 1.97E-04 | 1.34 | 1.97E-04 | 1.21 |
| Fumaric acid | | | 1.26E-07 | | 1.97E-04 | | 1.97E-04 | |
| Gluconic acid | 1.27E-04 | | 5.24E-08 | | 2.22E-04 | | 1.97E-04 | |
| Gluconic acid lactone | 1.88E-04 | | 3.05E-03 | | 3.29E-02 | | 1.03E-06 | |
| Glucose-6-phosphate | 1.35E-02 | 1.92 | | 1.15 | 3.89E-03 | 1.36 | 1.97E-04 | 1.75 |
| Glyceric acid | 1.97E-04 | 1.17 | 1.97E-04 | | | | 2.81E-06 | |
| Glycerol-1-phosphate | 1.85E-02 | | 2.27E-02 | | | | 7.84E-03 | |
| Homoserine | 1.97E-04 | 1.42 | | | 3.97E-03 | | | |
| Hypoxanthine | | | | | 4.02E-02 | 1.34 | 3.89E-03 | 1.39 |
| Lanosterol | 2.09E-03 | 1.19 | 1.97E-04 | 1.47 | 1.09E-03 | | 8.29E-04 | |
| Leucine | 3.05E-03 | | 2.37E-03 | | 1.97E-04 | 1.18 | 1.97E-04 | |
| Linoleic acid | 2.37E-03 | 1.17 | | | 6.29E-04 | | 1.97E-04 | 1.22 |
| Lysine | | | | | 4.02E-02 | 1.45 | 9.50E-04 | 1.56 |
| Malic acid | 2.44E-02 | | | | 1.84E-03 | | 1.97E-04 | |
| Mannitol / xylitiol | 4.45E-04 | | 1.10E-02 | | 9.14E-08 | | 1.82E-05 | |
| Melezitose | 1.97E-04 | 1.31 | | | 4.74E-04 | | 1.97E-04 | 1.31 |
| Methionine | 3.08E-03 | | | | 1.97E-04 | | 1.97E-04 | |
| Oleic acid | | | | | 2.15E-04 | | 1.97E-04 | |
| Ornithine | | | 4.02E-02 | 1.48 | 1.35E-02 | 1.49 | 1.97E-04 | 1.59 |
| Phenylalanine | 4.94E-03 | | 2.37E-03 | 1.17 | 1.97E-04 | 1.04 | 1.97E-04 | 1.11 |
| Phosphoenolpyruvic acid | | | | | 1.35E-02 | 1.34 | 1.35E-02 | 1.18 |
| P-hydroxyphenyllactic acid | | | | | 2.27E-02 | 1.29 | 9.79E-03 | |
| Pipecolic acid | | | | | | | | |
| Proline | 1.97E-04 | 1.74 | 1.97E-04 | 1.19 | 1.97E-04 | 1.49 | 1.97E-04 | |
| Putrescine | 7.84E-03 | | | | 8.68E-04 | | | |
| Ribose-5-phosphate | 1.35E-02 | 1.81 | 4.02E-02 | 1.47 | 3.89E-03 | 1.46 | 1.97E-04 | 1.72 |
| Serine | | | 1.96E-04 | | 1.97E-04 | | 1.97E-04 | |
| Squalene | | | 1.79E-08 | | 2.05E-05 | | 4.74E-04 | |
| Sucrose | | | | | | | 5.42E-03 | |
| Threonine | 1.32E-07 | 1.05 | 1.96E-05 | | 1.97E-04 | 1.17 | 4.07E-06 | |
| Tyrosine | | | | | 6.29E-04 | 1.01 | 1.97E-04 | 1.14 |
| Uracil | | | | | 3.05E-03 | | 1.97E-04 | |
| Urea | 4.74E-04 | | 1.38E-04 | | 1.41E-02 | | 1.39E-03 | |
| Uridine-5-monophosphate | 1.35E-02 | 1.69 | 9.50E-04 | 1.97 | 3.89E-03 | 1.31 | 1.97E-04 | 1.72 |

*Lm*: *L. major* Friedlin*; Lt*: *L. tarentolae* LV-414; *Lb*: *L. braziliensis* M2904; *Li*: *L. infantum* PP75; *La*: *L. amazonensis* PH8; VIP: variable importance in projection.

TABLE III

Summary of statistically significant metabolites that were responsible for enriching KEGG pathways

| Metabolic pathways | *Lt* x *Li* | *Lt* x *Lm* | *Lt* x *La* | *Lt* x *Lb* |
|---|---|---|---|---|
| ABC transporters | | | Putrescine; leucine, mannitol, threonine. | |
| Biosynthesis of unsaturated fatty acids | Eicosanoic acid; behenic acid; linoleic acid; oleic acid. | Eicosanoic acid; behenic acid; linoleic acid; oleic acid. | | |
| Citrate cycle (TCA cycle) | Citric acid; malic acid; phosphoenolpyruvic acid; fumaric acid. | Citric acid; malic acid; phosphoenolpyruvic acid; fumaric acid. | | |
| Glycine, serine and threonine metabolism | | | Glyceric acid; homoserine; threonine. | |
| Glyoxylate and dicarboxylate metabolism | | 3-phosphoglyceric acid; citric acid; glyceric acid; malic acid. | Citric acid; glyceric acid; malic acid. | |
| Pentose-phosphate pathway | 6-phosphogluconic acid; gluconic acid; gluconic acid lactone; ribose-5-phosphate. | 6-phosphogluconic acid; gluconic acid; gluconic acid lactone; glyceric acid; ribose-5-phosphate. | 6-phosphogluconic acid; gluconic acid; gluconic acid lactone; glyceric acid; ribose-5-phosphate. | 6-phosphogluconic acid; gluconic acid; gluconic acid lactone; glyceric acid; ribose-5-phosphate. |
| Steroid biosynthesis | Ergosterol; cholesterol; squalene; lanosterol. | Ergosterol; cholesterol; squalene; lanosterol. | Ergosterol; lanosterol; cholesterol. | Ergosterol; lanosterol; squalene. |

KEGG: Kyoto Encyclopedia of Genes and Genomes; *Lm*: *Leishmania major* Friedlin; *Lt*: *L. tarentolae* LV-414; *Lb*: *L. braziliensis* M2904; *Li*: *L. infantum* PP75; *La*: *L. amazonensis* PH8.

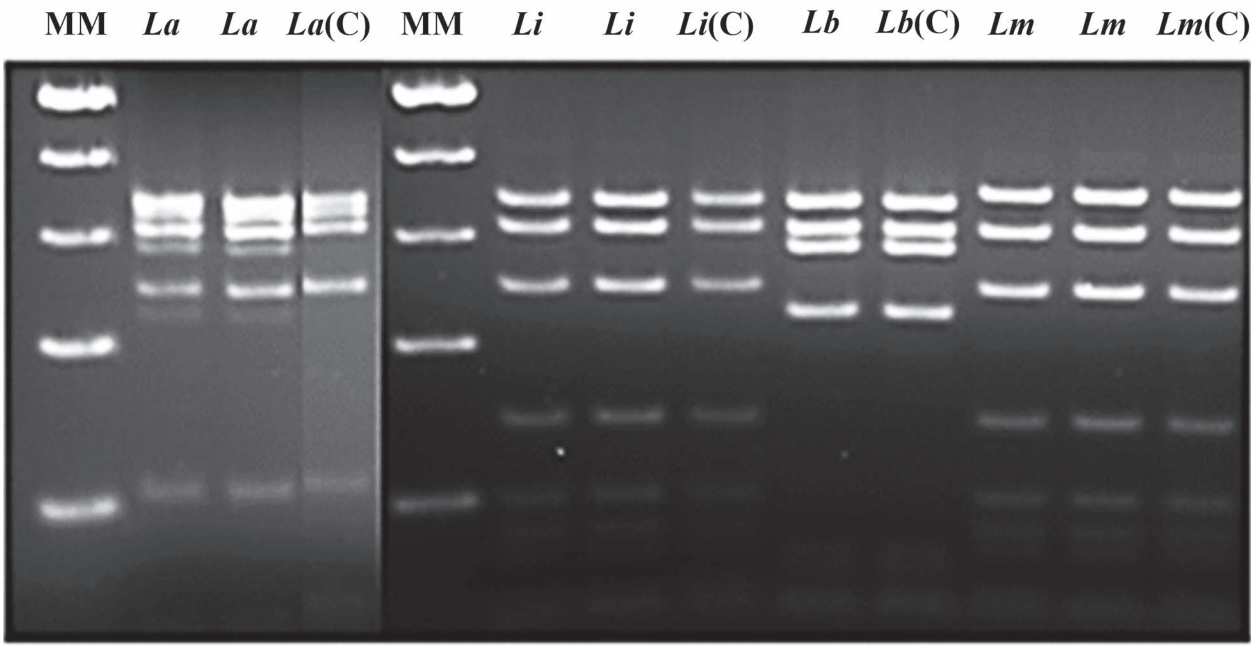


Fig. 1: polymerase chain reaction (PCR) restriction profiles from the strains used in this study with HaeIII. Control strains: *Leishmania amazonensis* (La) (IFLA/BR/67/PH8), *L. braziliensis* (Lb) (MHOM/BR/75/M2903), *L. infantum* (Li) (MHOM/BR/74/PP75), *L. guyanensis* (Lg) (MHOM/BR/75/M4147) and *L. major* (Lm) (MHOM/SU/1973/5-ASKH). MM: molecular weight size marker and (C): control.

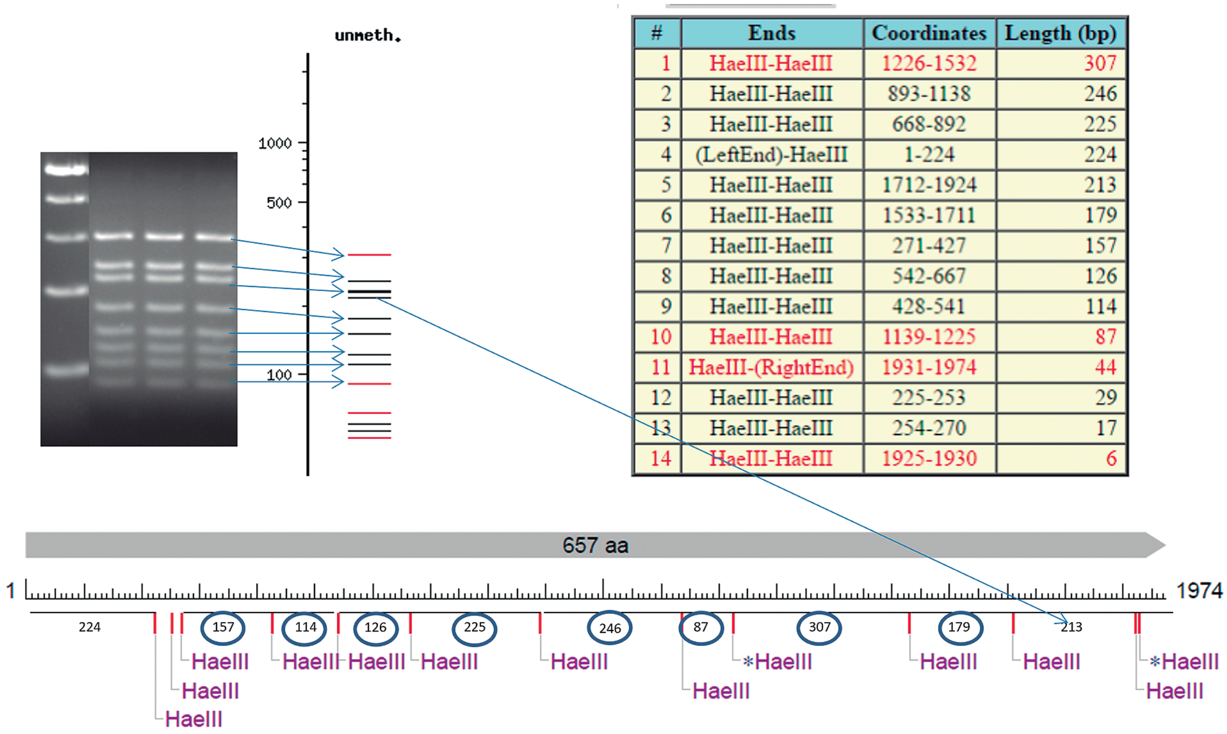


| # | Ends | Coordinates | Length (bp) |
|---|---|---|---|
| 1 | HaeIII-HaeIII | 1226-1532 | 307 |
| 2 | HaeIII-HaeIII | 893-1138 | 246 |
| 3 | HaeIII-HaeIII | 668-892 | 225 |
| 4 | (LeftEnd)-HaeIII | 1-224 | 224 |
| 5 | HaeIII-HaeIII | 1712-1924 | 213 |
| 6 | HaeIII-HaeIII | 1533-1711 | 179 |
| 7 | HaeIII-HaeIII | 271-427 | 157 |
| 8 | HaeIII-HaeIII | 542-667 | 126 |
| 9 | HaeIII-HaeIII | 428-541 | 114 |
| 10 | HaeIII-HaeIII | 1139-1225 | 87 |
| 11 | HaeIII-(RightEnd) | 1931-1974 | 44 |
| 12 | HaeIII-HaeIII | 225-253 | 29 |
| 13 | HaeIII-HaeIII | 254-270 | 17 |
| 14 | HaeIII-HaeIII | 1925-1930 | 6 |

Fig. 2: comparison of *Leismania tarentolae* heat shock protein 70 (hsp70) profile to *in silico* restriction analysis deposited in GenBank (AY423268).[51]

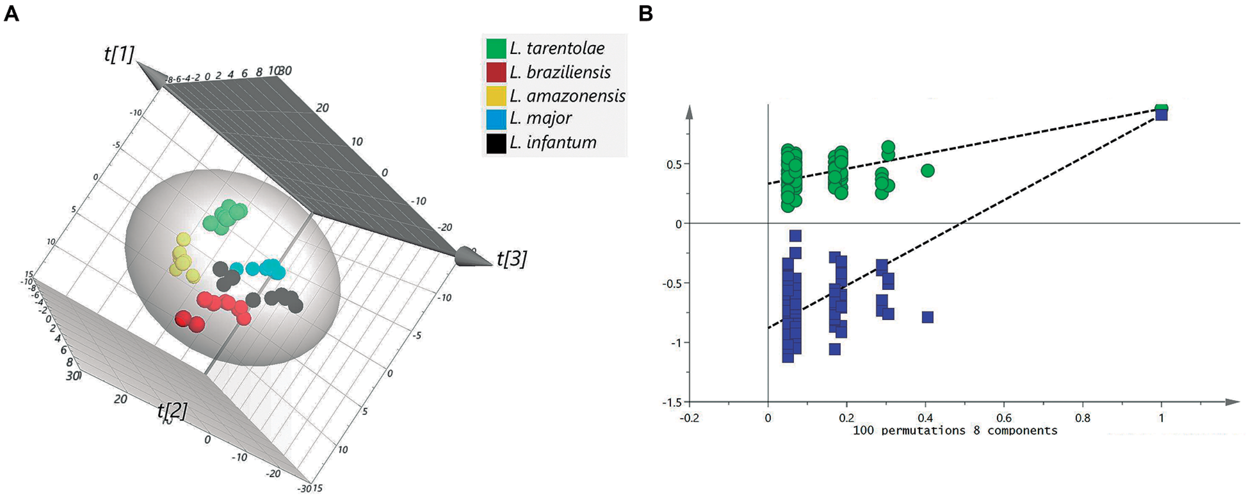


Fig. 3: partial least squares-discriminant analysis (PLS-DA) of metabolomics data. (A) PLS-DA model was built with total molecular features, separating samples in five groups: *Leishmania braziliensis* (red), *L. major* (blue), *L. amazonensis* (yellow), *L. infantum* (black) and *L. tarentolae* (green). Quality parameters of the analysis: R2X = 0.883, R2Y = 0.962, Q2X = 0.903; CV-ANOVA: p-value = $5.37x10^{-32}$. (B) PLS-DA validation model by permutation test (100-iterations), intercepts: R2 = 0.0, 0.333; Q2 = 0.0, -0.881.

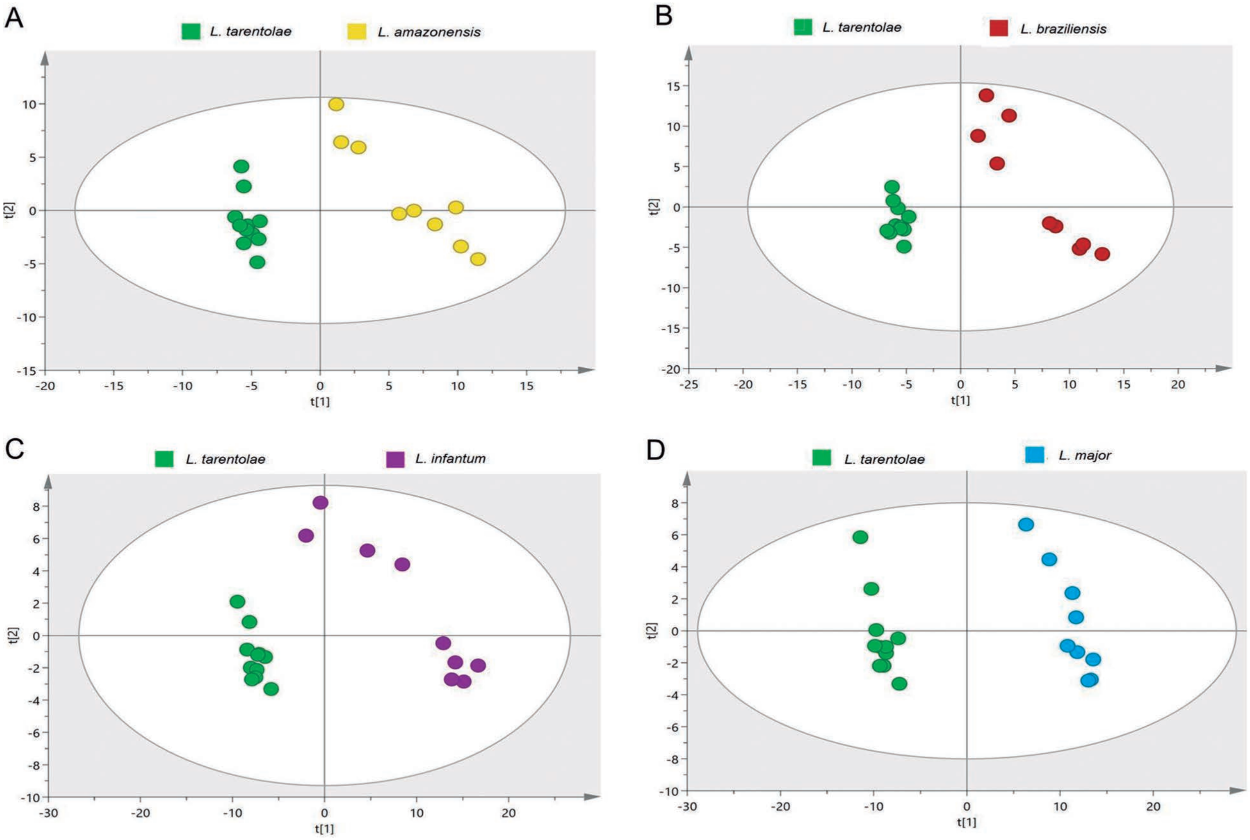


Fig. 4: partial least squares-discriminant analysis (PLS-DA) built with total identified metabolites, comparing species in pairs. (a) *Leishmania tarentolae* (green) and *L. amazonensis* (yellow). R2X = 0.721, R2Y = 0.991 and Q2X = 0.944; (b) *L. tarentolae* and *L. braziliensis* (red). R2X = 0.784, R2Y = 0.991 and Q2X = 0.966 (c) *L. tarentolae* and *L. infantum* (purple). R2X = 0.873, R2Y = 0.995 and Q2X = 0.976 (d) *L. tarentolae* and *L. major* (blue). R2X: 0.85, R2Y = 0.993 and Q2X = 0.986.
